# The prevalence and risk factors of work related musculoskeletal disorders among electronics manufacturing workers: a cross-sectional analytical study in China

**DOI:** 10.1186/s12889-022-14952-6

**Published:** 2023-01-03

**Authors:** Feng Yang, Niu Di, Wei-wei Guo, Wen-bin Ding, Ning Jia, Hengdong Zhang, Dongxia Li, Dayu Wang, Rugang Wang, Danying Zhang, Yongquan Liu, Bo Shen, Zhong-xu Wang, Yan Yin

**Affiliations:** 1 Department of Occupational Health and Poisoning Control, Shanghai Municipal Center for Disease Control and Prevention, Shanghai, 200336 China; 2 Department of Occupational Protection and Ergonomics, National Institute of Occupational Health and Poison Control, Chinese Center for Disease Control and Prevention, Beijing, 100050 China; 3 Jiangsu Provincial Center for Disease Control and Prevention, Nanjing, Jiangsu China; 4 Guizhou Province Occupational Disease Prevention and Control Hospital, Guiyang, Guizhou China; 5 Tianjin Occupational Disease Prevention and Control Hospital, Tianjin, China; 6 Beijing Center for Disease Control and Prevention, Beijing, China; 7 Guangdong Province Hospital for Occupational Disease Prevention and Treatment, Guangzhou, Guangdong China; 8 Institute of Occupational Medicine of Jiangxi, Nanchang, Jiangxi China; 9 Fuzhou Center for Disease Control and Prevention, Fuzhou, Fujian China

**Keywords:** Work-related musculoskeletal disorders, Electronics manufacturing industry, Epidemiology

## Abstract

**Objectives:**

To describe the prevalence of self-reported musculoskeletal disorders among workers in the electronics manufacturing industry and to investigate the relations between work-related musculoskeletal disorders (WMSDs) and work-related variables.

**Methods:**

An interview-based questionnaire survey was carried out in thirty electronics manufacturing factories in China in 2018. The prevalence of WMSDs was estimated using the modified Nordic Musculoskeletal Questionnaire (NMQ). A multivariate logistic regression model was applied to evaluate the effects of risk factors on WMSDs on multiple body parts.

**Results:**

The 12-month prevalence of WMSDs among participants was 40.6%, and the common body sites affected were the neck (26.8%), shoulder (22.8%), upper back (14.9%), and lower back (14.8%). The results of logistic regression showed that female adults, > 5 job tenure and work-related factors (including awkward posture, lifting or carrying weights, excessive repetition, prolonged sitting, monotonous work and working under conditions of cold or temperature variations) led to a higher risk of WMSDs on most body parts. Upper back, wrist/hand and elbow pain levels were significantly higher for workers with vibration. However, more frequently, physical exercise was a protective factor against WMSDs on most body parts except the upper back, leg and knee.

**Conclusions:**

The study indicates a high prevalence of musculoskeletal pain among the electronics manufacturing industry in China. Different personal and work factors are related to the occurrence of WMSD on different body parts. Preventive measures should be implemented based on the characteristics of WMSD in the electronic manufacturing industry. Furthermore, the training and intervention guidance of ergonomic hazards in the workplace need to be strengthened by understanding the impact of bad posture, avoiding long-term sitting posture and increasing physical activities.

## Introduction

Work-related musculoskeletal disorders (WMSDs) have become one of the main reasons for the decline of the labour force of the occupational population. Research on the disease burden in 2019 shows that disability-adjusted life years caused by lower back pain rank fourth in the world among the 25–49 year population [1]. In 2016, globally, an estimated 76.1 (66.3–86.3) million DALYs were attributable to the included occupational risk factors; among them, 20.3% DALYs were attributable to ergonomic exposure, which ranked second behind injury risk factors (28.2%); furthermore, this trend is still increasing [2]. WMSDs and their related factors have become a global public health problem and are worthy of attention and research.

WMSDs are multifactorial in nature, as there are several factors that may contribute to developing these problems, including biomechanical, psychosocial, organizational, individual and environmental factors [3, 4]. To date, most studies have shown that WMSDs on various body parts have different risk factors. When these single independent factors were combined, the risks of WMSDs were affected [5–7].

The electronics industry is one of the largest global industries and is known for its rapid technological innovation, global competition, and labour intensity [8, 9]. High ergonomic risks include high load, repetition, awkward posture, and monotonous work in the workplace. The prevalence of WMSDs in the electronics industry was 35.7%~80.5% on different body parts in previous studies [10–12].

A review of the literature shows that there is limited research on the occurrence of musculoskeletal symptoms and their contributing risk factors among electronic manufacturing workers. To date, a few previous studies have focused on single or several similar factories, such as a thin film transistor liquid crystal display (TFT-LCD) manufacturing factory [8], and electronic part processing factories [9, 10] as well as certain workers, such as women [11]. The results of these previous studies only reflect the occurrence of WMSDs in certain enterprises or working populations and lack the prevalence and distribution characteristics of WMSDs in electronics industry industries.

Therefore, the current large-scale epidemiological survey on the electronics industry in thirty factories in China, including different types of factories, was conducted to determine the prevalence of and risk factors for work-related musculoskeletal disorders among electronics manufacturing workers. This study also enhances the body of research on MSDs and has the potential to inform work practices in similar occupational groups.

## Subjects and methods

### Participants

This cross-interventional study was conducted in 2018 and comprised 7307 electronics manufacturing workers from 30 electronics manufacturing enterprises in North, South, East and Southwest China. The enterprises were randomly selected by the enterprise scale and include semiconductor manufacturing, electronic chip manufacturing, computer manufacturing operations and so on.

The participants were recruited via cluster sampling; they worked various jobs across 40 departments, including 14 types of work, e.g., assembly, quality inspection, monitoring, welding, packaging, and wafer fabrication. They reported sitting or standing for a prolonged time, performed the repetitive operations or worked in awkward postures and had at least one year of work experience in their current job. The exclusion criteria were as follows: congenital spinal malformation or musculoskeletal disorders caused by trauma, infectious diseases, malignant tumours and other nonwork-related factors. All participants read and signed the informed consent approved by the China CDC ethics committee.

### The questionnaire

The data were collected using the electronic questionnaire system of the Chinese Version of the Musculoskeletal Disorders Questionnaire, which has been tested for reliability and validity for the Chinese population [13, 14] provided by the Chinese Center for Disease Control and Prevention. The questionnaire contains 3 parts: demographic characteristics, musculoskeletal symptoms and work-related factors.

The demographic characteristics included gender, age, job tenure, body mass index (BMI, weight/height^2^), and educational level, as well as individual habits such as weekly amount of physical activity. The work-related factors included awkward posture, lifting or carrying weights (> 5 kg), excessive repetition, daily working hours, sitting for a long period at work, vibration and work environment involving cold or cool wind.

The definition of WMSDs most commonly employed by the National Institute of Occupational Safety and Health (NIOSH) included the following criteria [15]: (1) discomfort within the past year; (2) discomfort began after employment in the current job; (3) no prior accident or sudden injury (affecting focal area of discomfort); and (4) episodes of discomfort occur monthly or, if not every month, at least exceeding a weeklong period of discomfort.

### Statistical analysis

All study variables were selected from the questionnaire responses. Some of them were coded as dummy variables (‘‘No” or ‘‘Yes, 0 and 1, respectively), and others were classified into more categories, as shown in Table 2.

Descriptive analyses, such as frequency distribution and the significance of crude associations between the outcome variables (WMSDs in neck, shoulder, upper back, lower back, elbow, wrist/hand, hip/thigh, knee, and ankle/foot), were tested with chi-square tests. The association between the outcome variable and independent variables was explored by multivariate logistic regression analysis, and the crude odds ratio (COR) was computed with a 95% CI.

All statistical analyses were performed using SPSS version 21.0 (SPSS Inc., Chicago, IL, USA), and a *P* value of less than 0.05 was considered statistically significant.

## Results

### Incidences of WMSDs in different body parts

Table 1 indicates the prevalence of WMSDs during the last 12 months among the workers in different parts of the body. The total prevalence of WMSDs was 40.2% among the workers, mainly involving the neck (26.8%), shoulder (22.8%), upper back (14.9%), and lower back (14.8%).

**Table 1:** Prevalence of WMSDs in different body parts in the workers

Body parts	Positive case (n)	Prevalence (%)
Neck	1959	26.81
Shoulder	1667	22.81
Upper back	1088	14.89
Low back	1078	14.75
Elbow	486	6.65
Wrist/hand	827	11.32
Hip/Thigh	665	9.10
Knee	536	7.34
Ankle/ foot	742	10.15
Total	2964	40.56

### Demographic characteristics analysis

A total of 3334 male (45.6%) and 3973 female (54.4%) workers were included, and their ages ranged from 16 to 62 years (mean 32.36 ± 7.39 years). A total of 5144 (70.40%) of the participants were 35 years old or younger, and 2163 (29.60%) were over 35 years old. The working duration ranged from 1 to 40 years (mean 4.66 ± 4.57 years). A majority of the respondents (5150, 70.48%) had less than 5 years of experience in the electronics industry. The body mass index values ranged from 14.19 to 50.34 kg/m^2^ (mean 22.8 ± 4.75 kg/m^2^). A total of 4616 (63.17%) of the participants had a normal BMI, ranging from 18.5 to 24.9 kg/m^2^, while 536 (7.43%) were classified as obese (BMI greater than 28 kg/m^2^). With respect to education, 1789 (24.48%) respondents attended junior college or above, 3558 (48.69%) respondents had completed their senior high school education, and 1960 (26.82%) respondents only attained a junior middle school education or below. Regarding exercise habits, 1759 (24.07%) respondents never exercised, 4272 (58.46%) exercised occasionally, and 1276 (17.46) exercised more than twice a month.

For most body parts, WMSDs were significantly associated with the participant’s sex (except the hip/thigh and knee), age (elbow, wrist/hand, hip/thigh and ankle/foot), job tenure (except the ankle or foot), and BMI (lower back, elbow, knee and ankle/foot) (Table 2). It is worth noting that as exercise frequency increased, the occurrence of WMSDs decreased significantly on most body parts (except the knee). The prevalence of upper back, wrist/hand, knee, ankle/foot, elbow, and hip/thigh pain was significantly associated with participant education.

**Table 2:** Prevalence and association of work-related musculoskeletal disorders to demographics among electronics manufacturing workers

	n	Neck (%)	Shoulder (%)	Upper back (%)	Lower back (%)	Elbow (%)	Wrist /hand (%)	Thigh (%)	Knee (%)	Ankle/ foot (%)
Gender										
Male	3334	18.75^c^	14.37^c^	11.01^c^	11.07^c^	5.58^c^	8.16^c^	8.55	7.5	11.28^b^
Female	3973	33.58	29.9	18.15	17.85	7.55	13.97	9.56	7.2	9.21
Education level										
Junior middle school or below	1960	26.53	23.62	14.08^c^	14.85	7.19^c^	12.50^c^	9.44^c^	7.35	10.05^b^
Senior high or secondary school	3558	27.07	23.19	16.72	15.54	7.39	12.06	9.95	7.81	11.07
Junior college	1118	26.21	21.47	13.24	13.6	5.37	9.39	8.68	6.71	9.57
Bachelor degree or above	671	27.27	20.72	10.28	12.22	3.28	7.15	4.32	5.81	6.56
Age (years old)										
< 26	1344	22.77^c^	18.97^c^	12.57^a^	11.68^b^	6.99	11.46	8.78	7.22^c^	10.79
26–35	3800	26.84	22.34	15.21	15.24	6.34	10.82	8.95	6.13	10
> 35	2163	29.26	26.03	15.77	15.81	6.98	12.11	9.57	9.52	10.03
Job tenure (years)										
< 2	3335	23.15^c^	19.37^c^	13.49^c^	13.31^c^	6.69^a^	11.39^c^	8.67^b^	6.84^c^	10.16
2 ~ 5	1815	25.67	21.27	12.78	12.95	5.45	9.04	7.82	5.73	9.04
> 5	2157	33.43	29.44	18.82	18.5	7.6	13.12	10.85	9.46	11.08
Physical exercise										
Never	1759	27.97^c^	23.82^c^	16.66^c^	16.43^c^	6.77^c^	10.92^c^	10.29^a^	7.28	11.94^c^
Occasionally	4272	27.67	23.74	14.86	15.15	7.37	12.57	9.32	7.61	10.42
2–3 times/month	343	30.03	23.03	15.74	13.99	5.54	11.37	7.29	8.75	9.33
1–2 times/week	566	20.14	17.49	11.48	9.89	3.18	5.65	6.36	4.95	6.36
More than 3 times/week	367	18.53	15.26	11.17	10.35	4.09	7.36	6.81	6.81	5.18
BMI (kg/m^2^, NA = 65)										
< 18.5	678	24.63	19.03	14.31	11.50^b^	6.64^b^	11.8	8.26	7.23^b^	9.59^b^
18.5–23.9	4616	27.45	23.2	14.62	14.49	6.24	10.88	8.75	6.65	9.55
24–27.9	1412	25.85	23.23	15.51	15.86	6.8	11.9	9.84	9.07	10.62
≥ 28	536	27.61	23.88	16.42	19.03	10.07	13.43	11.38	8.96	14.74

^a^*P* < 0.05

^b^*P* < 0.01

^c^*P* < 0.001

### Work-related factor analysis

Regarding work-related factors (Table 3), the majority of study participants (3038, 41.57%) performed their task in an awkward posture, 3912 (53.54%) were often/always lifting or carrying weights (> 5 kg), and 3789 (51.85%) worked with excessive repetition. Respondents who met these criteria were more likely to have WMSDs than their counterparts (*P* < 0.05). Working under conditions of cold or temperature variations was also associated with WMSDs in all nine body parts. A total of 75.65% of the workers worked often/always sitting for a long time. More often, sitting workers had significantly more WMSDs in the neck, shoulder, upper and lower backs than those who reported never or rarely sitting (*p* < 0.001). However, subjects who reported more sitting had a significantly lower prevalence of hip/thigh, knee and ankle or foot pain than those who reported not sitting (*p* < 0.001). A total of 1535 (21.70%) respondents reported working often with vibration tools, which was also associated with WMSDs (except neck, shoulder and lower back), *p* < 0.001. Taking turns with colleagues was a protective factor against WMSDs in the neck, shoulder, upper back, and lower back, *p* < 0.05.

**Table 3:** Prevalence and association of work-related musculoskeletal disorders to work factors among electronics manufacturing workers

	n	Neck (%)	Shoulder (%)	Upper back (%)	Lower back (%)	Elbow (%)	Wrist /hand (%)	Thigh (%)	Knee (%)	Ankle /foot (%)
Awkward posture										
Never/fewer	4269	20.10^c^	17.29^c^	10.00^c^	10.24^c^	3.49^c^	6.54^c^	5.65^c^	4.66^c^	6.42^c^
Often/Always	3038	36.24	30.58	21.76	21.1	11.09	18.04	13.96	11.09	15.4
Lifting or carrying weights										
Never/fewer	3395	23.36^c^	19.82^c^	11.66^c^	11.16^c^	4.45^c^	8.57^c^	6.01^c^	5.21^c^	6.69^c^
Often/Always	3912	29.81	25.41	17.69	17.87	8.56	13.7	11.78	9.18	13.16
Excessive repetition										
Never/fewer	3518	17.60^c^	14.41^c^	8.19^c^	8.58^c^	3.30^c^	4.83^c^	5.49^c^	4.83^c^	7.73^c^
Often/Always	3789	35.37	30.61	21.11	20.48	9.77	17.34	12.46	9.66	12.4
Sitting for long period at work										
Never/fewer	1779	19.90^c^	14.95^c^	11.92^c^	12.42^c^	6.46	9.78^a^	11.58^c^	10.68^c^	16.81^c^
Often/Always	5528	29.03	25.34	15.85	15.5	6.71	11.81	8.3	6.26	8.01
Vibration										
Never/fewer	5772	26.61	22.61	14.21^c^	14.21^a^	5.53^c^	10.05^c^	8.33^c^	6.53^c^	8.84^c^
Often/Always	1535	27.56	23.58	17.46	16.81	10.88	16.09	11.99	10.36	15.11
Doing the same job every day										
Never/fewer	992	16.33^c^	14.21^c^	8.27^c^	8.57^c^	4.23^c^	5.75^c^	4.44^c^	4.44^c^	6.25^c^
Often/Always	6315	28.46	24.16	15.93	15.72	7.03	12.19	9.83	7.79	10.77
Take turns with colleagues										
Never/fewer	3608	29.43^c^	25.39^c^	15.80^a^	15.74^a^	6.76	11.5	9.34	7.29	9.17^b^
Often/Always	3699	24.25	20.3	14	13.79	6.54	11.14	8.87	7.38	11.11
Working environment involves cold or cool wind										
Never/fewer	5946	26.10^b^	22.12^b^	13.99^c^	13.67^c^	5.67^c^	10.38^c^	8.12^c^	6.58^c^	9.10^c^
Often/Always	1361	29.9	25.86	18.81	19.47	10.95	15.43	13.37	10.65	14.77

^a^*P* < 0.05

^b^*P* < 0.01

^c^*P* < 0.001

### Risk factor modelling by multivariate logistic regression

Variables with *p* values < 0.05 in the bivariate analysis were subsequently included in a multivariate logistic regression analysis to identify independent predictors of work-related musculoskeletal disorders (Table 4). In multivariate analysis, the risk of WMSDs among females was 1.96 (1.72 ~ 2.23), 1.67 (1.48 ~ 1.89), 1.55 (1.30 ~ 1.84), 1.51 (1.30 ~ 1.76), 1.49 (1.28 ~ 1.73) and 1.38 (1.12 ~ 1.69) times higher than that in males in the shoulder, neck, wrist/hand, upper back, lower back and elbow, respectively. The risk also increased with job tenure (except in the foot). Compared with workers with a job tenure of two years or less, workers who had a job tenure of 5 years or more had 1.59 (1.39 ~ 1.82) and 1.57 (1.38 ~ 1.79) times as many problems with shoulder and neck pain, respectively.

**Table 4:** Multivariate logistic regression analysis of factors influencing WMSDs in different body parts among electronics manufacturing workers

	Neck *OR*(95%*CI*)	Shoulder *OR*(95%*CI*)	Upper back *OR*(95%*CI*)	Lower back *OR*(95%*CI*)	Elbow *OR*(95%*CI*)	Wrist or hand *OR*(95%*CI*)	Hip/Thigh *OR*(95%*CI*)	Knee *OR*(95%*CI*)	Ankle/foot *OR*(95%*CI*)
**Gender**									
Male	1	1	1	1	1	1			
Female	1.67 (1.48 ~ 1.89)^c^	1.96 (1.72 ~ 2.23)^c^	1.51 (1.30 ~ 1.76)^c^	1.49 (1.28 ~ 1.73)^c^	1.38 (1.12 ~ 1.69)^b^	1.55 (1.30 ~ 1.84)^c^			
**Job tenure (years)**									
< 2	1	1	1	1	1	1	1	1	
2 ~ 5	1.11 (0.96 ~ 1.27)	1.06 (0.92 ~ 1.24)	0.89 (0.75 ~ 1.06)	0.92 (0.77 ~ 1.10)	0.78 (0.60 ~ 1.00)	0.74 (0.60 ~ 0.90)^b^	0.88 (0.71 ~ 1.09)	0.81 (0.62 ~ 1.04)	
> 5	1.57 (1.38 ~ 1.79)^c^	1.59 (1.39 ~ 1.82)^c^	1.37 (1.17 ~ 1.60)^c^	1.35 (1.15 ~ 1.58)^c^	1.10 (0.88 ~ 1.37)	1.09 (0.91 ~ 1.30)	1.33 (1.10 ~ 1.60)^b^	1.29 (1.02 ~ 1.62)^a^	
**Education level**									
Junior middle school or below			1				1		
Senior high school or technical secondary school			1.27 (1.08 ~ 1.50)^b^				0.97 (0.80 ~ 1.18)		
Junior college			1.15 (0.92 ~ 1.45)				0.94 (0.72 ~ 1.22)		
Bachelor degree or above			0.97 (0.72 ~ 1.31)				0.54 (0.36 ~ 0.82)^b^		
**BMI (kg/m2, NA = 65)**									
< 18.5				1	1				1
18.5–23.9				1.31 (1.01 ~ 1.69)	0.96 (0.68 ~ 1.34)				1.02 (0.77 ~ 1.35)
24–27.9				1.40 (1.05 ~ 1.87)	1.01 (0.69 ~ 1.48)				1.09 (0.79 ~ 1.49)
≥ 28				1.82 (1.30 ~ 2.53)^c^	1.65 (1.08 ~ 2.53)^a^				1.62 (1.13 ~ 2.33)^b^
**Physical exercise**									
Never	1	1		1	1	1			1
Occasionally	1.00 (0.88 ~ 1.14)	1.02 (0.89 ~ 1.18)		0.92 (0.79 ~ 1.08)^a^	1.16 (0.93 ~ 1.46)	1.26 (1.05 ~ 1.52)			0.88 (0.73 ~ 1.06)
2–3 times/month	1.25 (0.95 ~ 1.63)	1.09 (0.81 ~ 1.46)		0.93 (0.66 ~ 1.31)^a^	0.94 (0.56 ~ 1.56)	1.31 (0.89 ~ 1.91)			0.78 (0.52 ~ 1.18)
1–2 times/week	0.77 (0.60 ~ 0.98)^a^	0.81 (0.63 ~ 1.05)		0.63 (0.46 ~ 0.86)^b^	0.51 (0.30 ~ 0.86)^a^	0.59 (0.40 ~ 0.89)			0.52 (0.35 ~ 0.75)^c^
More than 3 times/week	0.66 (0.49 ~ 0.89)^b^	0.67 (0.49 ~ 0.93)^a^		0.70 (0.48 ~ 1.02)	0.79 (0.45 ~ 1.39)	0.88 (0.57 ~ 1.36)			0.51 (0.31 ~ 0.83)^b^
**Excessive repetition**									
Often/Always	1.89 (1.68 ~ 2.13)^c^	1.89 (1.67 ~ 2.15)^c^	2.15 (1.84 ~ 2.51)^c^	1.97 (1.69 ~ 2.29)^c^	2.26 (1.80 ~ 2.83)^c^	2.93 (2.43 ~ 3.53)^c^	1.93 (1.60 ~ 2.32)^c^	1.82 (1.49 ~ 2.22)^c^	1.45 (1.22 ~ 1.72)^c^
**Awkward posture**									
Often/Always	2.64 (2.22 ~ 3.14)^c^	2.27 (1.89 ~ 2.72)^c^	2.23 (1.84 ~ 2.70)^c^	2.24 (1.85 ~ 2.72)^c^	3.06 (2.41 ~ 3.88)^c^	2.43 (1.98 ~ 2.99)^c^	2.78 (2.24 ~ 3.44)^c^	2.89 (2.29 ~ 3.64)^c^	2.84 (2.29 ~ 3.51)^c^
**Lifting or carrying weights**									
Often/Always	1.26 (1.09 ~ 1.46)^b^	1.31 (1.13 ~ 1.53)^c^	1.47 (1.25 ~ 1.74)^c^	1.76 (1.49 ~ 2.07)^c^	1.30 (1.03 ~ 1.64)^a^	1.44 (1.20 ~ 1.74)^c^	1.22 (1.00 ~ 1.49)^a^	1.47 (1.19 ~ 1.81)^c^	1.59 (1.33 ~ 1.91)^c^
**Sitting for long period at work**									
Often/Always	2.02 (1.80 ~ 2.26)^c^	2.01 (1.78 ~ 2.27)^c^	1.67 (1.45 ~ 1.92)^c^	1.64 (1.42 ~ 1.90)^c^		1.40 (1.19 ~ 1.64)^c^	0.80 (0.68 ~ 0.95)^a^	0.72 (0.59 ~ 0.87)^c^	0.40 (0.33 ~ 0.47)^c^
**Doing the same job every day**									
Often/Always	1.37 (1.14 ~ 1.65)^b^	1.26 (1.03 ~ 1.54)^a^	1.34 (1.04 ~ 1.71)^a^	1.28 (1.00 ~ 1.63)^a^		1.36 (1.02 ~ 1.82)^a^	1.69 (1.22 ~ 2.33)^c^		1.52 (1.15 ~ 2.02)^b^
**Take turns with colleagues**									
Often/Always		0.88 (0.78 ~ 0.99)^a^							
**vibration**									
Often/Always			1.45 (1.13 ~ 1.86)^b^		1.89 (1.40 ~ 2.55)^c^	1.48 (1.14 ~ 1.94)^b^			
**Working environment involves cold or cool wind**									
Often/Always		1.22 (1.05 ~ 1.42)^b^	1.23 (1.04 ~ 1.45)^a^	1.36 (1.15 ~ 1.61)^c^	1.62 (1.30 ~ 2.02)^c^	1.34 (1.12 ~ 1.61)^b^	1.35 (1.12 ~ 1.64)^b^	1.35 (1.09 ~ 1.67)^b^	1.27 (1.05 ~ 1.53)^a^

^a^*P* < 0.05

^b^*P* < 0.01

^c^*P* < 0.001

Working with a strained posture was significantly associated with WMSDs in all nine body parts, especially in the elbow (OR: 3.06, 95% CI (0.41 ~ 3.88)), knee (OR: 2.89, 95% CI (2.29 ~ 3.64)), foot (OR: 2.84, 95% CI(2.29 ~ 3.51)), hip/thigh (OR: 2.78, 95% CI(2.24 ~ 3.44)) and neck (OR: 2.64, 95% CI (2.22 ~ 3.14)). People who lifted heavy weights (5 kg) often had more problems than those who lifted seldom or never. The impacts on the lower back (OR: 1.76, 95% CI (1.49 ~ 2.07)), foot (OR: 1.59, 95% CI (1.33 ~ 1.91)), and knee (OR: 1.47, 95% CI (1.19 ~ 1.81)) were more significant. As excessive repetition increased, pain increased significantly in most body parts, especially in the wrist/hand (OR: 2.93, 95% CI (2.43 ~ 3.53)), elbow (OR: 2.26, 95% CI (1.80 ~ 2.83)), and upper back (OR: 2.15, 95% CI (1.84 ~ 2.51)).

Sitting for a long time was another important risk factor for MSDs in the neck, shoulder, back and wrist/hand; however, the problems decreased significantly with prolonged sitting in the following body parts: foot (OR: 0.40, 95% CI (0.33 ~ 0.47)), knee (OR: 0.72 95% CI (0.59 ~ 0.87)) and hip/thigh (OR: 0.80,95% CI (0.68 ~ 0.95)). Working under conditions of cold or temperature variations was also associated with WMSDs in most body parts except the neck. Physical exercise has a significant protective effect against WMSDs in the foot, neck, shoulder, lower back, wrist/hand and elbow. Vibration was a risk factor for WMSDs in the upper back (OR: 1.89, 95% CI (1.40 ~ 2.55), wrist/hand (OR: 1.48, 95% CI (1.14 ~ 1.94) and upper back (OR: 1.45, 95% CI (1.13 ~ 1.86)). In addition to the above factors, education level is a risk factor for WMSDs in the hip/thigh and upper back. Lower back, foot and elbow problems were affected by BMI, such that the higher the BMI value was, the higher the prevalence of WMSDs.

## Discussion

In this study, we investigated the prevalence of work-related musculoskeletal disorders among electronics manufacturing workers in the last 12 months. The overall prevalence of WMSDs in our study was 40.6%, and the common body sites affected were the neck, shoulder, upper back and low back. The total prevalence is consistent with reports from a study in Beijing [9] but lower than the rates reported in Iran [10, 16], the USA [17], and Peninsular Malaysia [11]. This may be because the respondents’ compositions by age and gender were inconsistent in different studies. In this study, respondents are younger than those in other studies, and some studies only focus on female workers in electronic factories. There is another reason to note that the questionnaire instruments have not been standardized in these studies, and large differences in prevalence may reflect differences between questionnaires. However, it is worth noting that the main problems in the electronics manufacturing industry are concentrated in the neck, shoulder and upper back, which has been shown in several studies [11, 16]. A systematic review that selected and examined 30 references suggested that the prevalence of musculoskeletal symptoms among handicraft workers ranged between 38.5% and 100%, and the most commonly affected body areas were the neck, back, knees and upper limbs [3].

Our study analyses the complex interrelationship of demographic and work factors with WMSDs. In this study, we observed a significantly higher prevalence of the top four body part complaints among females (33.57%, 29.90%, 18.15%, 17.85%) than among males (18.75%, 14.38%, 11.01%, 11.07%). There were significant associations between the participant’s sex and the prevalences of WMSDs in the neck, shoulder, upper back and lower back. This result was consistent with the findings of other epidemiological studies [8, 18] since female workers tend to have a smaller body build and are less able to bear loads than male workers. Ostergren PO et al. indicated that the effect of psychosocial factors was more prominent in women, which could be a synergistic effect of psychosocial and biological factors in heightening the risk of developing shoulder and neck pain among women [19].

Furthermore, age was not associated with the prevalence of WMSDs in the regression model after considering other factors. Age was not an independent influencing factor but coexisted with other potential factors. A similar finding was reported by Bruno R [20]. However, work experience was associated with the prevalence of the four associated parts WMSDs, which has also been reported in previous studies in those body parts among bank staff [21]. Workers who had a long work duration (≥ 5 years of work experience) had a higher risk of developing WMSDs than those with less work experience. This might be because a long job tenure leads to more exposure to risk factors than a short job tenure. This means that work-related musculoskeletal disorder by its nature is cumulative trauma or repetitive strains that develop gradually as a result of overuse.

The most commonly reported biomechanical risk factors with at least reasonable evidence for causing WMSD include excessive repetition, awkward postures, and heavy lifting [22–24]. In this study, we found the same result: biomechanical risk factors were potential risk factors associated with the development of musculoskeletal symptoms in all nine body parts among electronic manufacturing workers. Tasks requiring repetitive movements are common in the electronic manufacturing industry. Repetitive movements of any muscle group will eventually cause fatigue in that group and result in tension in other muscles during execution of the task. When using the upper limbs in a repetitive manner, muscles in the shoulder, neck, and back are usually tensed and become fatigued during the course of movement.

Few studies have examined the relationship between working environment temperature change and WMSDs in the electronic manufacturing industry. A total of 27.4% of workers complained that the working environment was too cold in a study by Heng Leng Chee [11]. In this study, 18.56% of workers complained of working under conditions of cold or temperature variations. It was found that significant work under conditions of cold or temperature variations had positive contributions to the prevalence of WMSDs in most body parts. This is consistent with previous reports of mining [25] and cold store work [26]. The results indicated that musculoskeletal symptoms are more frequent in cold exposure than in normal temperature work. Most of the production workshops in the electronics industry are clean workshops, which have strict requirements on temperature, humidity and ventilation. The associations between clean workshops and musculoskeletal disorders need further research.

Sitting for a long time and monotonous work are the characteristics of assembly line workers in the electronic manufacturing industry. The present study also found that sitting for a long period at work and doing the same job every day were associated with higher odds of WMSDs in most body parts. An association between neck and shoulder pain and sitting was also found in Malaysian electronics factory workers [12] but not with other body parts. Whether prolonged sitting is a risk factor for lower back pain remains unclear. Previous research has indicated that prolonged sitting could increase the risk of lower back pain. Possible mechanisms mentioned are increased intradiscal pressure stiffness of the lumbar spine and reduced strength of the lower back muscles [27, 28]. However, a recent systematic review reported that poor sitting posture and lack of daily physical activities may be strong predictors of LBP caused by sitting, not only prolonged sitting [29].

Physical activity is considered an effective measure to prevent WMSDs in many studies [30, 31]. In our study, pain reports in most body parts (except the knee) decreased significantly with an increase in weekly exercise frequency. Physical activity is a protective factor for these body parts. PA helps to promote muscle activation patterns different from those imposed by work to restore the balance of spinal stability [29]. A multicentre randomized clinical trial study among Italian surgeons demonstrated the effectiveness of a global program based on the application of ergonomics in the operating room and specific physical exercises in the lower back [32]. Shariat et al. showed that exercise modification was more effective than ergonomic modification in the treatment of office workers with neck, shoulder, and lower back pain [33]. When working long hours, long sitting operations are very common in electronic manufacturing workers, and there is little time for sports after work, which is an effective way to organize employees to exercise/stretch in the workplace to prevent WMSDs.

## Limitations

This study had some limitations that should be acknowledged when interpreting the results. It is only a cross-sectional study, and it is difficult to determine causality. Retrospective analysis and its accuracy need to be verified by prospective studies. In addition, the relationship between psychological factors and WMSDs was not analysed in this study. Future research is needed to explore the effects of these risk factors on WMSDs.

## Conclusion

In summary, the prevalence of WMSDs was relatively high in electronic manufacturing workers in China, especially in the neck, shoulder and back. We found that excessive repetition, awkward posture, and heavy lifting were significant work-related risk factors for WMSDs in all nine body parts. In addition, working under conditions of cold or temperature variations is related to the occurrence of WMSDs (except in the neck), which was first discovered in the electronics industry. It is notable that sedentary work (75.65%) and a lack of exercise (82.54%) were common in this sample, thereby affecting the occurrence of WMSDs in the neck, shoulder and back. As a result, prevention and control programs in the workplace are necessary in the electronics industry, from engineering design, cognitive training, organizational management and individual behaviour to avoid or reduce the adverse technical factors in the work and reduce the occurrence of WMSDs.

## Data Availability

The datasets used and/or analyzed during the current study available from the corresponding author on reasonable request.

## References

[1] GBD 2019 Diseases and Injuries Collaborators (2020). Global burden of 369 diseases and injuries in 204 countries and territories, 1990–2019: a systematic analysis for the Global Burden of Disease Study 2019. Lancet.

[2] GBD 2016 Occupational Risk Factors Collaborators (2020). Global and regional burden of disease and injury in 2016 arising from occupational exposures: a systematic analysis for the Global Burden of Disease Study 2016. Occup Environ Med.

[3] Das D, Kumar A, Sharma M (2020). A systematic review of work-related musculoskeletal disorders among handicraft workers. Int J Occup Saf Ergon..

[4] Das B (2022). Ergonomic and psychosocial risk factors for low back pain among rice farmers in West Bengal, India. Work.

[5] Widanarko B (2014). The combined effect of physical, psychosocial/organisational and/or environmental risk factors on the presence of work-related musculoskeletal symptoms and its consequences. Appl Ergon.

[6] Keir PJ (2021). Relationships and mechanisms between occupational risk factors and distal upper extremity disorders. Hum Factors.

[7] Seidel DH (2019). Quantitative measures of physical risk factors associated with work-related musculoskeletal disorders of the elbow: a systematic review. Int J Environ Res Public Health.

[8] Chu P, Wang T, Guo YL. Work-related and personal factors in shoulder disorders among electronics workers: findings from an electronics enterprise in Taiwan. BMC Public Health. 2021;21(1). 10.1186/s12889-021-11572-4. 10.1186/s12889-021-11572-4 PMC8351339 34372812

[9] Wang S, Mamat N, Wang F (2019). Analyzing the potential category of occurrence pattern of musculoskeletal disorders among workers in an electronic parts processing factory. Chin Occup Med.

[10] Daneshmandi H (2019). An ergonomic intervention to relieve musculoskeletal symptoms of assembly line workers at an electronic parts manufacturer in Iran. Work.

[11] Chee HL, Rampal KG (2004). Work-related musculoskeletal problems among women workers in the semiconductor Industry in peninsular Malaysia. Int J Occup Environ Health.

[12] Maimaiti N (2019). Cervical musculoskeletal disorders and their relationships with personal and work-related factors among electronic assembly workers. J Saf Res.

[13] Yang L, Hildebrandt VH, Yu SH-f (2009). Study on musculoskeletal disease questionnaire. Ind Health Occup Dis.

[14] Zhang D, Lu L, Hu H (2020). Analysis of risk factors of multi-site work-related musculoskeletal disorders among workers in the industry of electronic equipment manufacturing. Chin Occup Med.

[15] Salvendy G (2012). Handbook of human factors and ergonomics.

[16] Aghilinejad M (2016). An ergonomic intervention to reduce musculoskeletal discomfort among semiconductor assembly workers. Work.

[17] Pocekay (1995). A cross-sectional study of musculoskeletal symptoms and risk factors in semiconductor workers. Am J Ind Med.

[18] Dianat I, Karimi MA (2016). Musculoskeletal symptoms among handicraft workers engaged in hand sewing tasks. J Occup Health.

[19] Östergren P (2005). Incidence of shoulder and neck pain in a working population: effect modification between mechanical and psychosocial exposures at work? Results from a one year follow up of the Malmö shoulder and neck study cohort. J Epidemiol Community Health.

[20] da Costa BR, Vieira ER (2010). Risk factors for work-related musculoskeletal disorders: a systematic review of recent longitudinal studies. Am J Ind Med.

[21] Etana G (2021). Prevalence of work related musculoskeletal disorders and associated factors among bank staff in Jimma city, Southwest Ethiopia, 2019: an institution-based cross-sectional study. J Pain Res.

[22] Das B (2020). Work-related injuries, postural stress, and musculoskeletal disorders among the railway track maintainers in India. Toxicol Ind Health.

[23] Jia N (2022). Prevalence and risk factors analysis for low back pain among occupational groups in key industries of China. BMC Public Health.

[24] Das B (2015). An evaluation of low back pain among female brick field workers of West Bengal, India. Environ Health Prev Med.

[25] Yong X (2020). A cross-sectional epidemiological survey of work-related musculoskeletal disorders and analysis of its influencing factors among coal mine workers in Xinjiang. Biomed Res Int.

[26] Pienimäki T (2002). Cold exposure and musculoskeletal disorders and diseases. A review. Int J Circumpolar Health.

[27] Murtezani A (2011). Prevalence and risk factors for low back pain in industrial workers. Folia Med (Plovdiv).

[28] Gupta N (2015). Is objectively measured sitting time associated with low back pain? A cross-sectional investigation in the NOMAD study. PLoS One.

[29] Wei M, Huang H (2020). Relationship between the low back pain and sedentary behaivor: a systematic review. China Sport Sci Technol.

[30] Ribas TM (2020). Impact of physical activity levels on musculoskeletal symptoms and absenteeism of workers of a metallurgical company. Rev Bras Med Trab.

[31] Van Eerd D (2016). Effectiveness of workplace interventions in the prevention of upper extremity musculoskeletal disorders and symptoms: an update of the evidence. Occup Environ Med.

[32] Giagio S (2019). A preventive program for work-related musculoskeletal disorders among surgeons. Ann Surg.

[33] Shariat A (2018). Effects of stretching exercise training and ergonomic modifications on musculoskeletal discomforts of office workers: a randomized controlled trial. Braz J Phys Ther.

